# Evils Arising from Daily Use of Astringent Mouth Washes

**Published:** 1879-05

**Authors:** J. N. Farrar

**Affiliations:** Brooklyn, N. Y.


### ARTICLE V.

### *Evils Arising from the Daily Use of Astringent Mouth-Washes.*

BY J. N. FARRAR, M. D., BROOKLYN, N. Y.

That the *habitual and daily use* of astringent washes is mischevious, I think must be apparent to nearly every honest dentist who understands the effects of astringent medicines upon living animal tissues.

The object of a mouth-wash is to assist nature in restoring inflamed or congested gums to health, and give "tone" to the mucus membrane generally. The daily use of some washes will keep the gums in apparent health, but it is very questionable whether they should be used so frequently. All ordinary and useful washes contain astringents, and are more or less valuable in assisting in restoring to health diseased soft tissues, but when this is accomplished their use should be discontinued. When the system gets out of order, people sometimes take arsenic to aid in the restoration of the general health, but having done this they do not or should not continue to take arsenic regularly.

In the careful observation for several years of the effects of astringent mouth-washes upon the gums, it has been noticed that while some cases have improved, most of them have been injured. The pleasant taste and sensation imparted to the mouth by their sweetened perfume is probably the principal cause of their popularity.

A manufacturer once said to me, innocently enough, "thousands of people have used my mouth-wash, and a large number come to me regularly for it, and say that they cannot get along without it. As soon as they are out of my mouth-wash *their gums become red and puffy*, and their oral secretions become *stringy and slimy*. My mouth-wash has become so popular that I have orders for it from all parts of the country. My customers write: Send me a dozen bottles or so; we cannot live without its daily use."

Stronger testimony to substantiate my views cannot often be given, for the reason that we have the truth given through the ignorance of the witness. One may eat opium regularly for a long time and feel compartively well, and believe the use thereof to be beneficial; but when the system has become habituated to these drugs and their use is discontinued abruptly, suffering ensues, and the condition of the system relaxes from its ordinary apparently healthy tone. In order to enjoy life after this habit has become established, *the victim is obliged to continue its daily use;* just so it is with astringent mouth-washes. They are very excellent, even indispensable, in curing diseased conditions of some mouths, but if continued daily for any considerable length of time after they have done their proper work, the condition of the mouth changes into a chronic abnormal state, sometimes called "second nature." The gums and mucus membrane call for them, and expect the stimulation which they give; *and their discontinuance may finally provoke a soft, congested, or other diseased condition of the gums and mouth.*

Astringents cause shrinkage of animal tissues, a necessary action to restore soft, spongy, inflamed, congested flesh to health, but the apparent effect of astringent mouth-washes upon healthy gums is to harden them unduly. They reduce mucus secretions below the normal and healthy quantity, and produce a hard, dry aspect which is often so beautiful that it is apt to be mistaken for perfect health. Under such circumstances the degree of its use necessary for healthfulness is not always easy to know. While some gums and sockets will stand this abuse, others with less powers of vital resistance, and especially those which have a diathesis predisposing them to premature absorption, will conclusively prove the error of the treatment by hastening the absorption and contraction of the margins of the gums, and consequent *exposure of the necks and roots of the teeth.* Especially is this so where the *stiff brush* is used with it. The young and vigorous in growth and health will, however, often withstand the abusive use of astringent gum-washes for a long time before its evil consequences will become apparent.

This is a secret of their extensive sale. But, generally, the direful effects come sooner or later, of hardened gums and exposed roots, with no remedy, a condition worse than decay of the teeth.

Tartar will cause absorption of the gums by inflammation, but astringents act differently, and their long continued use causes the life to more or less shrink out of the tissues, producing artificial atrophy, probably from the deprivation or reduction of healthy action in the secreting cells, by keeping them in this medical splint so long that they lose in a measure the power of their normal functions. There is a law with all secreting tissues which leads to atrophy if their natural functions are suspended for any considerable length of time. It is my opinion that astringent gum and tooth-washes, so called, should never be used in healthy mouths, and this is the expressed opinion of all the celebrated dentists who have spoken upon the matter in the American Dental Association since its organization.

Some dentists think differently, and are always talking about mouth-washes to their patients, especially those who are in the trade. Mouth-washes should be made to meet the necessities of each case or disease for which they are prescribed; and they should be discontinued by degrees, or gradually divested of their astringency when health is restored, for it should be borne in mind that the chief potency and value of any gum-wash are its astringent qualities. The honey, perfume, alcohol, water and soponacious ingredients, of which nearly all are largely composed, are of but little value above ordinary soap and water.

The safer plan is to discontinue the wash as soon as health is restored, just as in any given case we would discontinue any other medicine under such circumstances, and to use nothing but simple tooth powder made of precipitated or prepared chalk, pulverized cuttle-fish bone, myrrh, and, perhaps, orris root, bicarbonate soda, and the like, which generally is all that is necessary to keep gums and teeth clean and in good condition after they are once restored to health, unless tartar collects too rapidly, when nothing but instruments will be effective. Although some washes are useful at proper times, I do not hesitate to say that most if not all, of the popular gum and tooth-washes sold in the shops and by some dentists are injurious in the long run, because they are recommended for daily use, whether the mouth be diseased or not.

Are they preservative? As to their tooth-saving qualities I can probably illustrate best by saying that the family of the manufacturer of the most extensively advertised tooth-wash in the world, advertised upon nearly every fence, rock, and newspaper throughout the length and breadth of the land, have recently come under my observation, and I believe that I have never seen poorer teeth than they have or presenting a greater degree of destruction from decay, though they have used this famous wash daily for years.

Diseased gums are almost always the result of neglect of cleanliness of the teeth, but such a condition may be, possibly, one of the symptoms of an affection of the nares, throat or stomach, or the result of careless medication, such as from mercurial poison.

Very seldom, do I find unhealthy mouths or gums, where a soft tooth-brush and a properly prepared tooth powder have been in daily use.

A harmless mouth-wash may be made, however, perfumed and not astringent, for imparting a pleasant taste and odor to the breath, which may be used daily. Such wash may be an inducement to oral cleanliness with indifferent people, when simple water and powder would be insufficient.

No toilet, however, is complete without a proper mouth-wash and tooth powder (unmixed.) The powder should be used every day, the astringent only when the gums or secretions are out of order. When in doubt as to what constitutes an abnormal condition of the mouth and gums, the wash may be used once or twice a week without doing harm, and will probably be beneficial *if* there be softness or redness of the gums or mucous membrane, with tendency to bleeding, or slimy, stringy saliva, or a general bad taste in the mouth.

All hybrid mixtures of liquids and powders made of such things as alcohol, water, tincture of catechu, honey and tooth powder, like all panacea medicines, are cheaply made to sell at a large profit. Complication in medicines, though formerly practiced, is now an old custom only continued by quacks. The right medicine in the right place may be a blessing, but if used out of place may be worse than a humbug.

---

